# Single-State Multi-Party Quantum Key Agreement with Single-Particle Measurement

**DOI:** 10.3390/e27040405

**Published:** 2025-04-10

**Authors:** Hao Yang, Dunbo Cai, Ling Qian, Runqing Zhang, Songfeng Lu, Chengfu Sun

**Affiliations:** 1 Hubei Key Laboratory of Distributed System Security, Hubei Engineering Research Center on Big Data Security, School of Cyber Science and Engineering, Huazhong University of Science and Technology, Wuhan 430074, China; d202180586@hust.edu.cn (H.Y.); lusongfeng@hust.edu.cn (S.L.); 2 Center for Technology Research & Innovation, China Mobile (Suzhou) Software Technology Co., Ltd., Suzhou 215163, China; caidunbo@cmss.chinamobile.com (D.C.); qianling@cmss.chinamobile.com (L.Q.); zhangrunqing@cmss.chinamobile.com (R.Z.); 3 Shenzhen Huazhong University of Science and Technology Research Institute, Shenzhen 518063, China; 4 School of Computer Science and Engineering, Huaiyin Institute of Technology, Huai’an 223003, China

**Keywords:** quantum cryptography, quantum entangled states, quantum key agreement

## Abstract

In this study, we propose a single-state multi-party quantum key agreement (MQKA) protocol with single-particle measurement. Firstly, a single-state three-party quantum key agreement protocol with single-particle measurement is introduced, followed by a security analysis that validated its capability to resist potential internal and external attacks. Furthermore, we utilize multi-particle entangled states to present a multi-party version of the single-state multi-party quantum key agreement with single-particle measurement. In comparison to previous MQKA protocols, our approach presents the following advantages: it employs one kind of multi-particle entangled state as the quantum resource; eliminates the need for entanglement swapping techniques, unitary operations, or pre-shared keys between participants; uses only the X measurement basis and Z measurement basis; transmits fewer qubits; consumes fewer qubits; and has higher qubit efficiency.

## 1. Introduction

Quantum cryptography, with its theoretically unconditional security feature, has gradually become an important research area in the field of cryptography. Unlike classical cryptography, the security of quantum cryptography is based on the quantum no-cloning theorem and Heisenberg’s uncertainty principle. As a result, it has attracted significant attention from researchers. Quantum cryptography includes several research areas, such as quantum key distribution (QKD) [1,2,3,4], quantum secret sharing (QSS) [5,6,7], quantum secure direct communication (QSDC) [8,9,10], quantum private query (QPQ) [11,12,13,14], and quantum key agreement (QKA). Among these, quantum key agreement (QKA) requires each participant to equally contribute to the generation of the negotiated key, ensuring that any non-trivial subset of participants cannot independently determine the key. In 2004, Zhou et al. [15] innovatively applied quantum teleportation to key agreement, proposing the first quantum key agreement (QKA) protocol. However, Tsai et al. [16] identified a significant flaw in Zhou’s protocol where a participant could independently determine the negotiated key. Subsequently, Hsueh et al. [17] implemented a QKA protocol using maximally entangled states. Nevertheless, Hsueh et al.’s protocol was vulnerable to controlled-NOT attacks [18]. In 2011, Chong et al. [19] enhanced the security of Hsueh et al.’s protocol. In 2013, Shi et al. [20] extended the two-party QKA to be multi-party and proposed the first multi-party QKA protocol, which utilizes the Bell state as the quantum resource state and employs entanglement swapping technology. On this basis, Sun et al. [21] proposed an efficient quantum key agreement protocol based on commutative encryption. Wang et al. [22] proposed a multi-party semiquantum key agreement without entanglement. In addition to these efforts, numerous QKA protocols have been proposed, such as the schemes based on a single particle [23,24,25,26], the Bell state [27,28,29,30,31,32], and a multi-particle entangled state [33,34,35,36,37,38,39,40].

Resently, Xu et al. [41] proposed a single-state multi-party semiquantum key agreement protocol based on multi-particle entangled states which offers several key advantages: it uses only one kind of multi-particle entangled state as the initial quantum resource; it does not require pre-shared keys between different parties; and it eliminates the need for unitary operations or quantum entanglement swapping. Subsequently, Yang et al. [42] proposed an efficient single-state multi-party quantum key agreement which retains most of the advantages of Xu et al.’s scheme while achieving significant improvements in the number of quantum state transmissions, the number of qubits consumed, and qubit efficiency.

In this paper, a single-state three-party quantum key agreement protocol utilizing single-particle measurements is proposed. We conduct a detailed analysis demonstrating that our scheme can resist potential internal and external attacks. Furthermore, we extend the proposed single-state three-party quantum key agreement protocol to be N-party by using N-particle entangled states instead of three-particle entangled states as the quantum resource states. Compared to previous multi-party QKA schemes, our protocol retains most of the advantages presented in references [41,42] while only requiring the use of the *X* measurement basis and *Z* measurement basis. Additionally, our protocol further reduces the number of qubits transmitted and consumed, and enhances qubit efficiency.

The rest of the paper is organized as follows: In Section 2, we describe the single-state three-party quantum key agreement protocol in detail. Section 3 provides a security analysis of the protocol. In Section 4, we extend the proposed single-state three-party quantum key agreement protocol to be N-party. Section 5 discusses the performance of our scheme. Section 6 provides a conclusion.

## 2. The Proposed Single-State Three-Party Quantum Key Agreement with Single-Particle Measurement

Suppose $P_{1}$, $P_{2}$, and $P_{3}$ seek to establish a private key over a quantum channel, ensuring that each participant contributes equally to the generation of the key and that the key cannot be fully determined by any non-trivial subset of them. In the negotiation phase, the utilization of a hash function outputting m bits is necessary.

Step 1: $P_{1}$, $P_{2}$, and $P_{3}$ randomly generate the CHECK keys

(1) $$\begin{array}{c} {\bar{K}}_{P_{1}}=\left\{{\bar{K}}_{P_{1}}^{1},{\bar{K}}_{P_{1}}^{2},\ldots ,{\bar{K}}_{P_{1}}^{l}\right\}, \end{array}$$

(2) $$\begin{array}{c} {\bar{K}}_{P_{2}}=\left\{{\bar{K}}_{P_{2}}^{1},{\bar{K}}_{P_{2}}^{2},\ldots ,{\bar{K}}_{P_{2}}^{l}\right\}, \end{array}$$

(3) $$\begin{array}{c} {\bar{K}}_{P_{3}}=\left\{{\bar{K}}_{P_{3}}^{1},{\bar{K}}_{P_{3}}^{2},\ldots ,{\bar{K}}_{P_{3}}^{l}\right\}, \end{array}$$

and the INFO keys

(4) $$\begin{array}{c} K_{P_{1}}=\left\{K_{P_{1}}^{1},K_{P_{1}}^{2},\ldots ,K_{P_{1}}^{n}\right\}, \end{array}$$

(5) $$\begin{array}{c} K_{P_{2}}=\left\{K_{P_{2}}^{1},K_{P_{2}}^{2},\ldots ,K_{P_{2}}^{n}\right\}, \end{array}$$

(6) $$\begin{array}{c} K_{P_{3}}=\left\{K_{P_{3}}^{1},K_{P_{3}}^{2},\ldots ,K_{P_{3}}^{n}\right\}. \end{array}$$

Here, *l* represents the number of the CHECK particles, *n* represents the length of the negotiated key, and ${\bar{K}}_{P_{1}},{\bar{K}}_{P_{2}},{\bar{K}}_{P_{3}},K_{P_{1}},K_{P_{2}},K_{P_{3}}\in \left\{0,1\right\}$. They calculate the hash values $H\left(K_{P_{1}}\right),H\left(K_{P_{2}}\right),H\left(K_{P_{3}}\right)$ corresponding to the keys $K_{P_{1}},K_{P_{2}},K_{P_{3}}$ and announce the values.

Step 2: $P_{1}$ prepares l+n three-particle GHZ states $|GHZ\rangle =\frac{1}{\sqrt{2}}\left(|000\rangle +|111\rangle \right)$. For each GHZ state, $P_{1}$ keeps the first particle, and sends the second particle to $P_{2}$ and the third particle to $P_{3}$. We denote the first particle sequence as $S_{P_{1}}$, the second particle sequence as $S_{P_{2}}$, and the third particle sequence as $S_{P_{3}}$ as follows:

(7) $$\begin{array}{c} S_{P_{1}}=\left\{S_{P_{1}}^{1},S_{P_{1}}^{2},\ldots ,S_{P_{1}}^{l+n}\right\}, \end{array}$$

(8) $$\begin{array}{c} S_{P_{2}}=\left\{S_{P_{2}}^{1},S_{P_{2}}^{2},\ldots ,S_{P_{2}}^{l+n}\right\}, \end{array}$$

(9) $$\begin{array}{c} S_{P_{3}}=\left\{S_{P_{3}}^{1},S_{P_{3}}^{2},\ldots ,S_{P_{3}}^{l+n}\right\}. \end{array}$$

Step 3: After $P_{2}$ receives $S_{P_{2}}$ and $P_{3}$ receives $S_{P_{3}}$, $P_{1}$ randomly selects *l* particles as the CHECK particles, and informs $P_{2}$, $P_{3}$ of the positions of the CHECK particles. Here, the CHECK particles held by $P_{1},P_{2},P_{3}$ are denoted as $U_{P_{1}},U_{P_{2}},U_{P_{3}}$, respectively, as follows:

(10) $$\begin{array}{c} U_{P_{1}}=\left\{U_{P_{1}}^{1},U_{P_{1}}^{2},\ldots ,U_{P_{1}}^{l}\right\}, \end{array}$$

(11) $$\begin{array}{c} U_{P_{2}}=\left\{U_{P_{2}}^{1},U_{P_{2}}^{2},\ldots ,U_{P_{2}}^{l}\right\}, \end{array}$$

(12) $$\begin{array}{c} U_{P_{3}}=\left\{U_{P_{3}}^{1},U_{P_{3}}^{2},\ldots ,U_{P_{3}}^{l}\right\}. \end{array}$$

Then, $P_{1},P_{2},P_{3}$ announce the CHECK keys ${\bar{K}}_{P_{1}},{\bar{K}}_{P_{2}},{\bar{K}}_{P_{3}}$. They calculate

(13) $$\begin{array}{c} \bar{K}={\bar{K}}_{P_{1}}⊕{\bar{K}}_{P_{2}}⊕{\bar{K}}_{P_{3}}. \end{array}$$

In this equation, $\bar{K}=\left\{{\bar{K}}^{1},{\bar{K}}^{2},\ldots ,{\bar{K}}^{l}\right\}$. For the *q*-th CHECK particle, when the value of ${\bar{K}}^{q}$ is 0, $P_{i}$ uses basis *Z* to measure the particle $U_{P_{i}}^{q}$ and publishes the results, where $q\in \left\{1,2,\ldots ,l\right\}$. When the value of ${\bar{K}}^{q}$ is 1, $P_{i}$ uses basis *X* to measure the particle $U_{P_{i}}^{q}$ and publishes the results, where $q\in \left\{1,2,\ldots ,l\right\}$. Suppose that the measurement results in basis *Z* are $U_{zP_{i}}^{q}=\left\{+1,-1\right\}$, and in basis *X* are $U_{xP_{i}}^{q}=\left\{+1,-1\right\}$. According to the entanglement properties of the three-particle GHZ state and Equation (14),

(14) $$\begin{array}{c} \begin{array}{c} |GHZ\rangle =\frac{1}{\sqrt{2}}\left(|000\rangle +|111\rangle \right)\\ =\frac{1}{\sqrt{4}}\left(|+++\rangle +|+--\rangle +|-+-\rangle +|--+\rangle \right) \end{array}. \end{array}$$

$P_{i}$ can check the correctness of the measurement results. When the chosen basis is *Z*, the measurement results must satisfy $U_{zP_{1}}^{q}=U_{zP_{2}}^{q}=U_{zP_{3}}^{q}$. When the chosen basis is *X*, the measurement results must satisfy $\Pi_{i=1}^{3}U_{xP_{i}}^{q}=1$. If the error exceeds the threshold, the protocol aborts. The remaining particles serve as INFO particles $V_{P_{i}}=\left\{V_{P_{i}}^{1},V_{P_{i}}^{2},\ldots ,V_{P_{i}}^{n}\right\}$. $P_{i}$ measures the particles with basis *Z*.

Step 4: The measurement results are represented by $V_{zP_{i}}$. If the measurement result is $|0\rangle$, its value is taken as 0; if the measurement result is $|1\rangle$, its value is taken as 1. According to the entanglement properties of the three-particle GHZ state, we can obtain $V_{zP_{1}}=V_{zP_{2}}=V_{zP_{3}}$. $P_{1}$ calculates $E_{P_{1}}=V_{zP_{1}}⊕K_{P_{1}}$ and sends it to $P_{2}$, $P_{3}$. $P_{2}$ calculates $E_{P_{2}}=V_{zP_{2}}⊕K_{P_{2}}$ and sends it to $P_{1}$, $P_{3}$. $P_{3}$ calculates $E_{P_{3}}=V_{zP_{3}}⊕K_{P_{3}}$ and sends it to $P_{1}$, $P_{2}$.

Step 5: $P_{1}$ calculates $H\left(E_{P_{2}}⊕V_{zP_{1}}\right)$, $H\left(E_{P_{3}}⊕V_{zP_{1}}\right)$. If $H\left(E_{P_{2}}⊕V_{zP_{1}}\right)=H\left(K_{P_{2}}\right)$ and $H\left(E_{P_{3}}⊕V_{zP_{1}}\right)=H\left(K_{P_{3}}\right)$, $P_{1}$ will infer the final negotiated key

(15) $$\begin{array}{c} K=K_{P_{1}}⊕\left(E_{P_{2}}⊕V_{zP_{1}}\right)⊕\left(E_{P_{3}}⊕V_{zP_{1}}\right). \end{array}$$

$P_{2}$ calculates $H\left(E_{P_{1}}⊕V_{zP_{2}}\right)$, $H\left(E_{P_{3}}⊕V_{zP_{2}}\right)$. If $H\left(E_{P_{1}}⊕V_{zP_{2}}\right)=H\left(K_{P_{1}}\right)$ and $H\left(E_{P_{3}}⊕V_{zP_{2}}\right)=H\left(K_{P_{3}}\right)$, $P_{2}$ will infer the final negotiated key

(16) $$\begin{array}{c} K=K_{P_{2}}⊕\left(E_{P_{1}}⊕V_{zP_{2}}\right)⊕\left(E_{P_{3}}⊕V_{zP_{2}}\right). \end{array}$$

$P_{3}$ calculates $H\left(E_{P_{1}}⊕V_{zP_{3}}\right)$, $H\left(E_{P_{2}}⊕V_{zP_{3}}\right)$. If $H\left(E_{P_{1}}⊕V_{zP_{3}}\right)=H\left(K_{P_{1}}\right)$ and $H\left(E_{P_{2}}⊕V_{zP_{3}}\right)=H\left(K_{P_{2}}\right)$, $P_{3}$ will infer the final negotiated key

(17) $$\begin{array}{c} K=K_{P_{3}}⊕\left(E_{P_{1}}⊕V_{zP_{3}}\right)⊕\left(E_{P_{2}}⊕V_{zP_{3}}\right). \end{array}$$

**Figure 1:**
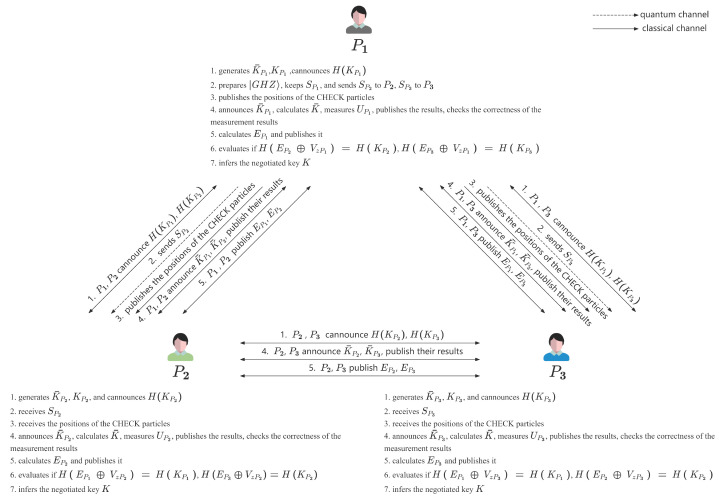
The flowchart depicts the mechanism of the proposed protocol.

As shown in Figure 1, the flowchart depicts the mechanism of the proposed MQKA protocol.

## 3. Security Analysis

This section will discuss the security of the proposed protocol. Without loss of generality, we analyze the security of the proposed three-party QKA protocol. The security analysis encompasses both internal attacks and external attacks.

### 3.1. External Attack

In the proposed three-party protocol, in order to obtain the negotiated key, an external attacker may launch typical attacks during the transmission of quantum states, including the Trojan horse attack, the intercept–resend attack, the entangle–measure attack, and the measure–resend attack.

#### 3.1.1. The Trojan Horse Attack

In our scheme, since each particle is transmitted only once, our three-party QKA protocol is immune to the delay-photon Trojan horse attack [43] and the invisible eavesdropping Trojan horse attack [44].

#### 3.1.2. The Intercept–Resend Attack

The external attacker may intercept the particles $P_{1}$ transmitted and resend the fake states to $P_{2}$, $P_{3}$. Without loss of generality, suppose that Eve intercepts the particles $S_{P_{3}}$ and sends the fake particles to $P_{3}$. Taking the *q*-th fake CHECK particle ${\tilde{U}}_{P_{3}}^{q}$($|0\rangle$) as an example, the *q*-th system state can be described as

(18) $$\begin{array}{c} \begin{array}{c} \tilde{\Psi }=\frac{1}{\sqrt{2}}\left(|00\rangle +|11\rangle \right)|0\rangle \\ =\frac{1}{\sqrt{4}}\left(|+++\rangle +|++-\rangle +|--+\rangle +|---\rangle \right) \end{array}. \end{array}$$

Firstly, we consider the scenario where the measurement basis is *Z*. When the measurement results of $U_{P_{1}}^{q},U_{P_{2}}^{q},{\tilde{U}}_{P_{3}}^{q}$ are $|000\rangle$, we can obtain $U_{zP_{1}}^{q}=U_{zP_{2}}^{q}={\tilde{U}}_{zP_{3}}^{q}$. Eve is capable of evading eavesdropping detection.

When the measurement results of $U_{P_{1}}^{q},U_{P_{2}}^{q},{\tilde{U}}_{P_{3}}^{q}$ are $|110\rangle$, we can obtain $U_{zP_{1}}^{q}=U_{zP_{2}}^{q}\neq {\tilde{U}}_{zP_{3}}^{q}$. Eve can be detected by the eavesdropping detection.

Secondly, we consider the scenario where the measurement basis is *X*. When the measurement results of $U_{P_{1}}^{q},U_{P_{2}}^{q},{\tilde{U}}_{P_{3}}^{q}$ in $|+++\rangle$ or $|--+\rangle$, we can obtain $U_{xP_{1}}^{q}\cdot U_{xP_{2}}^{q}\cdot {\tilde{U}}_{xP_{3}}^{q}=1$. Eve is capable of evading eavesdropping detection.

When the measurement results of $U_{P_{1}}^{q},U_{P_{2}}^{q},{\tilde{U}}_{P_{3}}^{q}$ in $|++-\rangle$ or $|---\rangle$, we can obtain $U_{xP_{1}}^{q}\cdot U_{xP_{2}}^{q}\cdot {\tilde{U}}_{xP_{3}}^{q}=-1$. Eve is unable to evade eavesdropping detection.

Therefore, for each CHECK particle, Eve is capable of evading eavesdropping detection with the probability of

(19) $$\begin{array}{c} \begin{array}{c} \frac{1}{2}\times \frac{1}{2}+\frac{1}{2}\times \left(\frac{1}{4}+\frac{1}{4}\right)=\frac{1}{2} \end{array}. \end{array}$$

For all *l* CHECK particles, Eve can be detected with the probability of $1-{\left(\frac{1}{2}\right)}^{l}$.

#### 3.1.3. The Entangle–Measure Attack

The external attacker may perform the entangle–measure attack. Without loss of generality, suppose that Eve intercepts the particles $P_{1}$ transmitted to $P_{2}$ ($P_{3}$), and performs unitary operation $U_{E}$ on the intercepted particles. If this attack does not introduce any errors, the system state of Eve’s probe should be independent of the measurement results of $S_{P_{1}},S_{P_{2}},S_{P_{3}}$. The effect of $U_{E}$ on $|0\rangle$ and $|1\rangle$ can be described as

(20) $$\begin{array}{c} \begin{array}{c} U_{E}\left(|0\rangle |E\rangle \right)=\varepsilon_{00}|0\rangle |e_{00}\rangle +\varepsilon_{01}|1\rangle |e_{01}\rangle \end{array}, \end{array}$$

(21) $$\begin{array}{c} \begin{array}{c} U_{E}\left(|1\rangle |E\rangle \right)=\varepsilon_{10}|0\rangle |e_{10}\rangle +\varepsilon_{11}|1\rangle |e_{11}\rangle \end{array}. \end{array}$$

In this equation, $|e_{00}\rangle$, $|e_{01}\rangle$, $|e_{10}\rangle$, and $|e_{11}\rangle$ are the probe state of Eve on $U_{E}$, and ${\left|\varepsilon_{00}\right|}^{2}+{\left|\varepsilon_{01}\right|}^{2}=1$, ${\left|\varepsilon_{10}\right|}^{2}+{\left|\varepsilon_{11}\right|}^{2}=1$. The global state of the system can be described as

(22) $$\begin{array}{c} \begin{array}{c} U_{E}\left({|GHZ\rangle }_{P_{1}P_{2}P_{3}}|E\rangle \right)\\ =U_{E}\left(\frac{1}{\sqrt{2}}\left({|0\rangle }_{P_{1}}{|0\rangle }_{P_{2}}{|0\rangle }_{P_{3}}+{|1\rangle }_{P_{1}}{|1\rangle }_{P_{2}}{|1\rangle }_{P_{3}}\right)|E\rangle \right)\\ =\frac{1}{\sqrt{2}}\left\{\begin{array}{c} {|0\rangle }_{P_{1}}\left(\varepsilon_{00}{|0\rangle }_{P_{2}}|e_{00}\rangle +\varepsilon_{01}{|1\rangle }_{P_{2}}|e_{01}\rangle \right)\left(\varepsilon_{00}{|0\rangle }_{P_{3}}|e_{00}\rangle +\varepsilon_{01}{|1\rangle }_{P_{3}}|e_{01}\rangle \right)+\\ {|1\rangle }_{P_{1}}\left(\varepsilon_{10}{|0\rangle }_{P_{2}}|e_{10}\rangle +\varepsilon_{11}{|1\rangle }_{P_{2}}|e_{11}\rangle \right)\left(\varepsilon_{10}{|0\rangle }_{P_{2}}|e_{10}\rangle +\varepsilon_{11}{|1\rangle }_{P_{2}}|e_{11}\rangle \right)+ \end{array}\right\}\\ =\frac{1}{\sqrt{2}}\left\{\begin{array}{c} \varepsilon_{00}\varepsilon_{00}{|0\rangle }_{P_{1}}{|0\rangle }_{P_{2}}{|0\rangle }_{P_{3}}|e_{00}\rangle |e_{00}\rangle +\varepsilon_{00}\varepsilon_{01}{|0\rangle }_{P_{1}}{|0\rangle }_{P_{2}}{|1\rangle }_{P_{3}}|e_{00}\rangle |e_{01}\rangle +\\ \varepsilon_{01}\varepsilon_{00}{|0\rangle }_{P_{1}}{|1\rangle }_{P_{2}}{|0\rangle }_{P_{3}}|e_{01}\rangle |e_{00}\rangle +\varepsilon_{01}\varepsilon_{01}{|0\rangle }_{P_{1}}{|1\rangle }_{P_{2}}{|1\rangle }_{P_{3}}|e_{01}\rangle |e_{01}\rangle +\\ \varepsilon_{10}\varepsilon_{10}{|1\rangle }_{P_{1}}{|0\rangle }_{P_{2}}{|0\rangle }_{P_{3}}|e_{10}\rangle |e_{10}\rangle +\varepsilon_{10}\varepsilon_{11}{|1\rangle }_{P_{1}}{|0\rangle }_{P_{2}}{|1\rangle }_{P_{3}}|e_{10}\rangle |e_{11}\rangle +\\ \varepsilon_{11}\varepsilon_{10}{|1\rangle }_{P_{1}}{|1\rangle }_{P_{2}}{|0\rangle }_{P_{3}}|e_{11}\rangle |e_{10}\rangle +\varepsilon_{11}\varepsilon_{11}{|1\rangle }_{P_{1}}{|1\rangle }_{P_{2}}{|1\rangle }_{P_{3}}|e_{11}\rangle |e_{11}\rangle \end{array}\right\} \end{array}, \end{array}$$

In this equation, the subscripts $P_{1}$, $P_{2}$, and $P_{3}$ denote the particles from $S_{P_{1}}$, $S_{P_{2}}$, and $S_{P_{3}}$, respectively. If Eve does not introduce any errors during the eavesdropping check by participants, the measurement results of $P_{1}$, $P_{2}$, and $P_{3}$ should be the same. Thereby, Eve’s attack $U_{E}$ should be satisfied with the conditions

(23) $$\begin{array}{c} \begin{array}{c} U_{E}\left({|GHZ\rangle }_{P_{1}P_{2}P_{3}}|E\rangle \right)\\ =\frac{1}{\sqrt{2}}\left(\varepsilon_{00}\varepsilon_{00}{|0\rangle }_{P_{1}}{|0\rangle }_{P_{2}}{|0\rangle }_{P_{3}}|e_{00}\rangle |e_{00}\rangle +\varepsilon_{11}\varepsilon_{11}{|1\rangle }_{P_{1}}{|1\rangle }_{P_{2}}{|1\rangle }_{P_{3}}|e_{11}\rangle |e_{11}\rangle \right) \end{array}. \end{array}$$

Based on Equation (22), we can infer

(24) $$\begin{array}{c} \begin{array}{c} \varepsilon_{00}\varepsilon_{01}|e_{00}\rangle |e_{01}\rangle +\varepsilon_{01}\varepsilon_{00}|e_{01}\rangle |e_{00}\rangle +\varepsilon_{01}\varepsilon_{01}|e_{01}\rangle |e_{01}\rangle +\\ \varepsilon_{10}\varepsilon_{10}|e_{10}\rangle |e_{10}\rangle +\varepsilon_{10}\varepsilon_{11}|e_{10}\rangle |e_{11}\rangle +\varepsilon_{11}\varepsilon_{10}|e_{11}\rangle |e_{10}\rangle =0 \end{array}. \end{array}$$

Here, 0 represents a column zero vector. Then, we can obtain

(25) $$\begin{array}{c} \begin{array}{c} \varepsilon_{00}\varepsilon_{01}+\varepsilon_{01}\varepsilon_{00}+\varepsilon_{01}\varepsilon_{01}+\varepsilon_{10}\varepsilon_{10}+\varepsilon_{10}\varepsilon_{11}+\varepsilon_{11}\varepsilon_{10}=0 \end{array}. \end{array}$$

Furthermore, we can obtain

(26) $$\begin{array}{c} \begin{array}{c} \varepsilon_{00}\varepsilon_{00}=\varepsilon_{11}\varepsilon_{11} \end{array}. \end{array}$$

(27) $$\begin{array}{c} \begin{array}{c} |e_{00}\rangle |e_{00}\rangle =|e_{00}\rangle |e_{11}\rangle \end{array}. \end{array}$$

Therefore, we can obtain

(28) $$\begin{array}{c} \begin{array}{c} U_{E}\left({|GHZ\rangle }_{P_{1}P_{2}P_{3}}|E\rangle \right)={|GHZ\rangle }_{P_{1}P_{2}P_{3}}|e_{00}\rangle |e_{00}\rangle \end{array}. \end{array}$$

Based on the aforementioned proof, in the absence of errors induced by Eve’s attack, the ultimate system state of Eve’s probe should be independent of the measurement results of the particles from $S_{P_{1}}$, $S_{P_{2}}$, and $S_{P_{3}}$. Therefore, Eve is unable to obtain any useful information about the measured particles. Once Eve acquires any useful information, Eve’s attack will be detected with a nonzero probability.

#### 3.1.4. The Measure–Resend Attack

The external attacker may intercept the particles $P_{1}$ transmitted, measure the particles, and resend the fake states to $P_{2}$, $P_{3}$. Without loss of generality, suppose that Eve intercepts the particles $S_{P_{3}}$, measures the intercepted particles with *Z* basis, and sends the fake particles to $P_{3}$. Clearly, the system state has a probability of $\frac{1}{2}$ for being $|000\rangle$ and a probability of $\frac{1}{2}$ for being $|111\rangle$.

Firstly, we consider the scenario where the system state is $|000\rangle$. When the measurement basis is *Z*, the measurement results of $U_{P_{1}}^{q}$, $U_{P_{2}}^{q}$, ${\tilde{U}}_{P_{3}}^{q}$ are $|000\rangle$, and we can obtain $U_{zP_{1}}^{q}=U_{zP_{2}}^{q}={\tilde{U}}_{zP_{3}}^{q}$. Eve is capable of evading eavesdropping detection.

When the measurement basis is *X* and the measurement results of $U_{P_{1}}^{q}$, $U_{P_{2}}^{q}$, ${\tilde{U}}_{P_{3}}^{q}$ in $|+++\rangle$, $|+--\rangle$, $|-+-\rangle$ or $|--+\rangle$, we can obtain $U_{xP_{1}}^{q}\cdot U_{xP_{2}}^{q}\cdot {\tilde{U}}_{xP_{3}}^{q}=1$. Eve is capable of evading eavesdropping detection.

When the measurement results of $U_{P_{1}}^{q}$, $U_{P_{2}}^{q}$, ${\tilde{U}}_{P_{3}}^{q}$ in $|++-\rangle$, $|+-+\rangle$, $|-++\rangle$ or $|---\rangle$, we can obtain $U_{xP_{1}}^{q}\cdot U_{xP_{2}}^{q}\cdot {\tilde{U}}_{xP_{3}}^{q}=-1$. Eve is unable to evade eavesdropping detection.

Secondly, we consider the scenario where the system state is $|111\rangle$. When the measurement basis is *Z*, the measurement results of $U_{P_{1}}^{q}$, $U_{P_{2}}^{q}$, ${\tilde{U}}_{P_{3}}^{q}$ are $|111\rangle$, and we can obtain $U_{zP_{1}}^{q}=U_{zP_{2}}^{q}={\tilde{U}}_{zP_{3}}^{q}$. Eve is capable of evading eavesdropping detection.

When the measurement basis is *X* and the measurement results of $U_{P_{1}}^{q}$, $U_{P_{2}}^{q}$, ${\tilde{U}}_{P_{3}}^{q}$ in $|+++\rangle$, $|+--\rangle$, $|-+-\rangle$ or $|--+\rangle$, we can obtain $U_{xP_{1}}^{q}\cdot U_{xP_{2}}^{q}\cdot {\tilde{U}}_{xP_{3}}^{q}=1$. Eve is capable of evading eavesdropping detection.

When the measurement results of $U_{P_{1}}^{q}$, $U_{P_{2}}^{q}$, ${\tilde{U}}_{P_{3}}^{q}$ in $|++-\rangle$, $|+-+\rangle$, $|-++\rangle$ or $|---\rangle$, we can obtain $U_{xP_{1}}^{q}\cdot U_{xP_{2}}^{q}\cdot {\tilde{U}}_{xP_{3}}^{q}=-1$. Eve is unable to evade eavesdropping detection.

Therefore, for each CHECK particle, Eve is capable of evading eavesdropping detection with the probability of

(29) $$\begin{array}{c} \begin{array}{c} \frac{1}{2}\times \left(\frac{1}{2}\times 1+\frac{1}{2}\times \left(\frac{1}{8}+\frac{1}{8}+\frac{1}{8}+\frac{1}{8}\right)\right)+\frac{1}{2}\times \left(\frac{1}{2}\times 1+\frac{1}{2}\times \left(\frac{1}{8}+\frac{1}{8}+\frac{1}{8}+\frac{1}{8}\right)\right)=\frac{3}{4} \end{array}. \end{array}$$

For all *l* CHECK particles, Eve can be detected with the probability of $1-{\left(\frac{3}{4}\right)}^{l}$.

### 3.2. Internal Attack

A secure quantum key agreement protocol is required to possess the fairness property, ensuring that all participants contribute equally to the negotiated key. In the proposed protocol, under the condition that all participants honestly execute the protocol prior to Step 4, they will be able to successfully obtain $V_{zP_{1}}=V_{zP_{2}}=V_{zP_{3}}$. In Step 4, $P_{i}$ calculates the value of $E_{P_{i}}=V_{zP_{i}}⊕K_{P_{i}}$ and publishes it to other participants, where $i\in \left\{1,2,3\right\}$. However, $P_{i}$ may announce the false value of ${\tilde{E}}_{P_{i}}$. Without loss of generality, suppose that $P_{1}$ intends to determine the negotiated key $\tilde{K}=\left\{{\tilde{K}}^{1},{\tilde{K}}^{2},\ldots ,{\tilde{K}}^{N}\right\}$ alone. In Step 4, $P_{2}$ publishes the value of $E_{P_{2}}=V_{zP_{2}}⊕K_{P_{2}}$, and $P_{3}$ publishes the value of $E_{P_{3}}=V_{zP_{3}}⊕K_{P_{3}}$. Based on the value of $E_{P_{2}}$ and $E_{P_{3}}$, $P_{1}$ can calculate the value of

(30) $$\begin{array}{c} \begin{array}{c} {\tilde{E}}_{P_{1}}=V_{zP_{1}}⊕\left(\tilde{K}⊕\left(E_{P_{2}}⊕V_{zP_{1}}\right)⊕\left(E_{P_{3}}⊕V_{zP_{1}}\right)\right) \end{array}. \end{array}$$

and announce the false value of ${\tilde{E}}_{P_{1}}$ to $P_{2}$, $P_{3}$, where ${\tilde{E}}_{P_{1}}=\left\{{\tilde{E}}_{P_{1}}^{1},{\tilde{E}}_{P_{1}}^{2},\ldots ,{\tilde{E}}_{P_{1}}^{n}\right\}$.

Based on the value of ${\tilde{E}}_{P_{1}}$, $P_{2}$ can infer the false value of

(31) $$\begin{array}{c} \begin{array}{c} \tilde{K}=K_{P_{2}}⊕\left({\tilde{E}}_{P_{1}}⊕V_{zP_{2}}\right)⊕\left(E_{P_{3}}⊕V_{zP_{2}}\right) \end{array}. \end{array}$$

$P_{3}$ can infer the false value of

(32) $$\begin{array}{c} \begin{array}{c} \tilde{K}=K_{P_{3}}⊕\left({\tilde{E}}_{P_{1}}⊕V_{zP_{3}}\right)⊕\left(E_{P_{2}}⊕V_{zP_{3}}\right) \end{array}. \end{array}$$

Nevertheless, as the hash value,

(33) $$\begin{array}{c} \begin{array}{c} H\left({\tilde{E}}_{P_{1}}⊕V_{zP_{2}}\right)\neq H\left(K_{P_{1}}\right) \end{array}. \end{array}$$

(34) $$\begin{array}{c} \begin{array}{c} H\left({\tilde{E}}_{P_{1}}⊕V_{zP_{3}}\right)\neq H\left(K_{P_{1}}\right) \end{array}. \end{array}$$

The dishonest behavior of $P_{1}$ will be detected by $P_{2}$, $P_{3}$.

Similarly, in the event that two dishonest participants collude to execute the cheating behavior, their cheating behavior will be exposed through the utilization of the hash function’s properties.

## 4. The Extension of the Proposed Scheme

Suppose $P_{1},P_{2},\ldots ,P_{N}$ seek to establish a private key over a quantum channel, ensuring that each participant contributes equally to the generation of the key and that the key cannot be fully determined by any non-trivial subset of them. In the negotiation phase, the utilization of a hash function outputting m bits is necessary.

Step 1: $P_{i}$ randomly generates the CHECK keys

(35) $$\begin{array}{c} \begin{array}{c} {\bar{K}}_{P_{i}}=\left\{{\bar{K}}_{P_{i}}^{1},{\bar{K}}_{P_{i}}^{2},\ldots ,{\bar{K}}_{P_{i}}^{l}\right\} \end{array}, \end{array}$$

and the INFO keys

(36) $$\begin{array}{c} \begin{array}{c} K_{P_{i}}=\left\{K_{P_{i}}^{1},K_{P_{i}}^{2},\ldots ,K_{P_{i}}^{n}\right\} \end{array}. \end{array}$$

Here, *l* represents the number of the CHECK particles, n represents the length of the negotiated key, and ${\bar{K}}_{P_{i}},K_{P_{i}}\in \left\{0,1\right\}$, $i\in \left\{1,2,\ldots ,N\right\}$. $P_{i}$ calculates the hash values $H\left(K_{P_{i}}\right)$ and announces the values.

Step 2: $P_{1}$ prepares l+n N-particle GHZ states ${|GHZ\rangle }_{N}=\frac{1}{\sqrt{2}}\left(|00,\ldots ,0\rangle +|11,\ldots ,1\rangle \right)$. For each GHZ state, $P_{1}$ keeps the first particle, and sends the t-th particle to $P_{t}$, where $t\in \left\{2,\ldots ,N\right\}$. We denote the *i*-th particle sequence as $S_{P_{i}}$ as follows:

(37) $$\begin{array}{c} \begin{array}{c} S_{P_{i}}=\left\{S_{P_{i}}^{1},S_{P_{i}}^{2},\ldots ,S_{P_{i}}^{l+n}\right\} \end{array}. \end{array}$$

Here, $i\in \left\{1,2,\ldots ,N\right\}$.

Step 3: After $P_{i}$ receives $S_{P_{i}}$, $P_{1}$ randomly selects *l* particles as the CHECK particles and informs other participants of the positions of the CHECK particles. Here, the CHECK particles respectively held by $P_{i}$ are denoted as $U_{P_{i}}$ as follows:

(38) $$\begin{array}{c} U_{P_{i}}=\left\{U_{P_{i}}^{1},U_{P_{i}}^{2},\ldots ,U_{P_{i}}^{l}\right\}. \end{array}$$

Then, $P_{i}$ announces the CHECK keys ${\bar{K}}_{P_{i}}$ and calculates

(39) $$\begin{array}{c} \begin{array}{c} \bar{K}={\bar{K}}_{P_{1}}⊕{\bar{K}}_{P_{2}}⊕\ldots ⊕{\bar{K}}_{P_{N}} \end{array}. \end{array}$$

Here, $\bar{K}=\left\{{\bar{K}}^{1},{\bar{K}}^{2},\ldots ,{\bar{K}}^{l}\right\}$. For the *q*-th CHECK particle, when the value of ${\bar{K}}^{q}$ is 0, $P_{i}$ uses the *Z* basis to measure the particle $U_{P_{i}}^{q}$ and publishes the measurement results, where $q\in \left\{1,2,\ldots ,l\right\}$. When the value of ${\bar{K}}^{q}$ is 1, $P_{i}$ uses the *X* basis to measure the particle $U_{P_{i}}^{q}$ and publishes the measurement results, where $q\in \left\{1,2,\ldots ,l\right\}$. Suppose that the measurement results in basis *Z* are $U_{zP_{i}}^{q}=\left\{+1,-1\right\}$ and in basis *X* are $U_{xP_{i}}^{q}=\left\{+1,-1\right\}$. According to the entanglement properties of the N-particle GHZ state and Equation (40),

(40) $$\begin{array}{c} \begin{array}{c} {|GHZ\rangle }_{N}=\frac{1}{\sqrt{2}}\left(\underset{N}{\underbrace{|00,\ldots ,0\rangle }}+\underset{N}{\underbrace{|11,\ldots ,1\rangle }}\right)\\ =\frac{1}{2^{\frac{N+1}{2}}}\sum_{\sigma_{1}\sigma_{2},\ldots ,\sigma_{N}}\left(|\sigma_{1}\sigma_{2},\ldots ,\sigma_{N}\rangle \right) \end{array}. \end{array}$$

In this equation, $\sigma_{i}\in \left\{+,-\right\}$, $i\in \left\{1,2,\ldots ,N\right\}$, $\sigma_{1}⊕\sigma_{2}⊕\cdots ⊕\sigma_{N}=0$. $P_{i}$ can check the correctness of the measurement results. When the chosen basis is *Z*, the measurement results must satisfy $U_{zP_{1}}^{q}=U_{zP_{2}}^{q}=\ldots =U_{zP_{N}}^{q}$. When the chosen basis is *X*, the measurement results must satisfy $\Pi_{i=1}^{N}U_{xP_{i}}^{q}=1$. If the error exceeds the threshold, the protocol aborts. The remaining particles serve as INFO particles $V_{P_{i}}=\left\{V_{P_{i}}^{1},V_{P_{i}}^{2},\ldots ,V_{P_{i}}^{n}\right\}$. $P_{i}$ measures the particles with the *Z* basis.

Step 4: The measurement results are represented by $V_{zP_{i}}$. If the measurement result is $|0\rangle$, its value is taken as 0; if the measurement result is $|1\rangle$, its value is taken as 1. According to the entanglement properties of the N-particle GHZ state, we can obtain $V_{zP_{1}}=V_{zP_{2}}=\ldots =V_{zP_{N}}$. $P_{i}$ calculates $E_{P_{i}}=V_{zP_{i}}⊕K_{P_{i}}$ and sends it to other participants.

Step 5: $P_{i}$ calculates $H\left(E_{P_{j}}⊕V_{zP_{i}}\right)$. If $H\left(E_{P_{j}}⊕V_{zP_{i}}\right)=H\left(K_{P_{j}}\right)$, $P_{i}$ will infer the final negotiated key

(41) $$\begin{array}{c} \begin{array}{c} K=\sum_{j=1}^{N}\left(E_{P_{j}}⊕V_{zP_{i}}\right)\left(mod\;2\right) \end{array}, \end{array}$$

In this equation, $i\in \left\{1,2,\ldots ,N\right\}$, $j\in \left\{1,2,\ldots ,N\right\}$. If any participant refuses *K* as the negotiated key, the protocol will be terminated and restarted.

## 5. Discussions

Reference [45] provides a definition of qubit efficiency,

(42) $$\begin{array}{c} \begin{array}{c} \eta =\frac{f}{q+c} \end{array}, \end{array}$$

In this equation, *q* is the number of transmitted qubits, *c* is the number of consumed classical bits, and *f* is the number of bits of the negotiated key. In the proposed protocol, $P_{1}$ prepares l+n N-particle entangled states, and it has $q=\left(l+n\right)N$. For the consumed classical bits, in order to announce the hash $H\left(K_{P_{i}}\right)$, the CHECK keys ${\bar{K}}_{P_{i}}$, the measurement results of $U_{P_{i}}$, and the ciphertext $E_{P_{i}}$, $P_{i}$ needs to spend *m*, *l*, *l*, and *n* classical bits, respectively, where *m* is the length of the hash function H(x). For *N* participants, the number of needed classical bits is $c=\left(2l+n+m\right)N$. The length of the negotiated key is *n*; therefore, f=n. Therefore, the qubit efficiency is $\eta =\frac{n}{\left(3l+2n+m\right)N}$. When *l* is the same as *n*, the qubit efficiency $\eta =\frac{n}{\left(5n+m\right)N}$.

In comparison to the previous MQKA protocols, as illustrated in Table 1, our protocol demonstrates great advantages. Our scheme retains most of the advantages of the schemes in [41,42] using one kind of multi-particle entangled state as the initial quantum resource, and eliminates the need for entanglement swapping techniques, unitary operations, or pre-shared keys between participants. In addition, our scheme uses only the X and Z bases for measurement, making it simpler and more practical than scheme [41], which uses GHZ and Z bases, and scheme [42], which uses X, Y, and Z bases. In terms of qubit transmission, our scheme and reference [42] require only one transmission from participant P1 to P2 (P3), while scheme [41] involves additional steps of reflecting or resending particles. Regarding qubit consumption, our scheme and reference [42] consume 2nN qubits, whereas reference [41] consumes $2^{N-1}n\left(3N-1\right)$ qubits. Moreover, our scheme achieves a qubit efficiency of $\frac{n}{5nN+mN}$, compared to $\frac{n}{2^{N-1}n\left(3N-1\right)+mN+nN}$ for reference [41] and $\frac{n}{6nN+mN}$ for reference [42]. These advantages make our scheme more efficient and practical for implementation.

## 6. Conclusions

In this paper, a single-state multi-party quantum key agreement (MQKA) protocol utilizing single-particle measurement is proposed. We introduce a single-state three-party quantum key agreement protocol with single-particle measurement followed by a comprehensive security analysis. Building on this foundation, we further extend this approach by employing multi-particle entangled states to develop a multi-party version of the single-state multi-party quantum key agreement with single-particle measurement. Specifically, it employs one kind of multi-particle entangled state as the quantum resource, eliminating the need for entanglement swapping techniques, unitary operations, or pre-shared keys between participants. Moreover, the protocol uses only the X measurement basis and Z measurement basis, which results in fewer qubits being transmitted and consumed, and higher qubit efficiency. While our method can be extended to a multi-party version, the preparation of multi-particle entangled states remains a significant challenge that warrants further investigation.

## Figures and Tables

**Table 1:** Comparison between our protocol and the previous MQKA protocols.

The MQKA Protocols	Quantum Resource States	Entanglement Swapping Technology	Unitary Operations	Pre-Shared Keys	Extra Qubits Transmission	Measurement Bases	Consumed Qubits	Quantum Efficiency
Protocol [41]	GHZ states	No	No	No	Yes	GHZ basis, *Z* basis	$2^{N-1}n$ (3N−1)	$\frac{n}{2^{N-1}n\left(3N-1\right)+mN+nN}$
Protocol [42]	GHZ states	No	No	No	No	*X* basis, *Y* basis, *Z* basis	2nN	$\frac{n}{6nN+mN}$
Our scheme	GHZ states	No	No	No	No	*X* basis, *Z* basis	2nN	$\frac{n}{5nN+mN}$

## Data Availability

Data are contained within the article.

## Abbreviations

The following abbreviations are used in this manuscript:

QKD	Quantum Key Distribution
QSS	Quantum Secret Sharing
QSDC	Quantum Secure Direct Communication
QPQ	Quantum Private Query
QKA	Quantum Key Agreement
